# Where do we go from here? Moving from systems-based practice process measures to true competency via developmental milestones

**DOI:** 10.3402/meo.v19.24441

**Published:** 2014-06-27

**Authors:** Johanna Martinez, Erica Phillips, Christina Harris

**Affiliations:** 1 Department of Medicine, Weill Medical College of Cornell University, New York, NY, USA;; 2 David Geffen School of Medicine, University of California, Los Angeles, CA, USA

**Keywords:** systems-based practice, core competencies, developmental milestones

## Abstract

For many educators it has been challenging to meet the Accreditation Council for Graduate Medical Education's requirements for teaching systems-based practice (SBP). An additional layer of complexity for educators is evaluating competency in SBP, despite milestones and entrustable professional activities (EPAs). In order to address this challenge, the authors present the results of a literature review for how SBP is currently being taught and a series of recommendations on how to achieve competency in SBP for graduate medical trainees with the use of milestones. The literature review included 29 articles and demonstrated that only 28% of the articles taught more than one of the six core principles of SBP in a meaningful way. Only 7% of the articles received the highest grade of A. The authors summarize four guiding principles for creating a competency-based curriculum that is in alignment with the Next Accreditation System (NAS): 1) the curriculum needs to include all of the core principles in that competency, 2) the objectives of the curriculum should be driven by clinical outcomes, 3) the teaching modalities need to be interactive and clinically relevant, and 4) the evaluation process should be able to measure competency and be directly reflective of pertinent milestones and/or EPAs. This literature review and the provided guiding principles can guide other residency educators in their development of competency-based curricula that meets the standards of the NAS.

For many educators, it has been challenging to meet the Accreditation Council for Graduate Medical Education's (ACGME) requirements for teaching the competency on systems-based practice (SBP) (1). Unveiled in 1999, the goal of the ACGME's Outcome Project was to move from basic knowledge and process measures to an accreditation system that focused on competencies. The multi-phase project began by inviting programs to define specific behaviors that would reflect the competency, as they pertained to that specific specialty. Phase II (2002–2006) of the project concentrated on refining the definitions of the competencies and their respective assessment tools. The goal of Phase III (2006–2011) was full integration of the competencies and their assessments into learning and clinical care. The Outcomes Project's goal was clear: ‘Residency programs are expected to phase in assessment tools that provide useful and increasingly valid, reliable, evidence that residents achieve competency-based educational objectives’ (2). Most recently, to assist educators in reaching the Outcomes Project's goal, the concepts of developmental milestones (3) and entrustable professional activities (EPAs) (4) have been established.

The educational outcomes are grounded in the six core competencies: 1) patient care; 2) medical knowledge; 3) practice-based learning and improvement (PBLI); 4) SBP; 5) professionalism; and 6) interpersonal skills and communication. Of these competencies, PBLI and SBP are the newest additions. SBP has six core principles and is defined as an ‘awareness of and responsiveness to larger context and system of health care and the ability to effectively call on system resources to provide care that is of optimal value’ (1). Specifically, all residents need to demonstrate the ability to:

1) work effectively in various health care delivery settings and systems relevant to their clinical specialty; 2) coordinate care within the health care system relevant to their clinical specialty; 3) incorporate considerations of cost awareness and risk benefit analysis in patient and/or population-based care, as appropriate; 4) advocate for quality patient care and optimal patient care systems; 5) work in interprofessional teams to enhance patient safety and improve patient care quality; and 6) participate in identifying system errors and implementing potential solutions.

The goals of the ACGME, as evidenced by Phase II of the Outcomes Project, are for residency programs to use dependable measures to assess residents’ competencies and, subsequently, to use those individual assessments to evaluate the educational effectiveness of the residency as a whole. As is clearly defined, these abilities are expected to be specific to each specialty (2) – and each competency should be sufficiently unique to merit its own category. Yet, data have shown that residencies are having difficulties measuring the individual competencies as independent constructs, and assessment tools often measure ‘behaviors that consistently map onto three or more of the general competencies’ (5). As a result, educators continue to struggle with the creation, definition, and categorization of six separate competencies. Furthermore, uncertainty continues to persist as to which assessment tools are most valid. Because true competency extends far beyond simple knowledge, educators are looking for measures that can assess ‘authentic’ behaviors of real-life clinical context. To date, only a ‘few models offer clear and direct routes’ for competency assessments without having measurement pitfalls (1).

Despite often being educators’ lowest priorities, this challenge especially exists for SBP and PBLI. Indeed, a survey of family medicine program directors rated SBP as the lowest importance of all the competencies, with 70% reporting no method to evaluate SBP (6). A major challenge is that SBP is truly a skill-based activity that cannot be validly captured with simple knowledge-based assessments. In response to these and other similar educational challenges, the ACGME developed 142 discrete curricular milestones that map to the six specific core competencies (3).

Even with the development of various educational constructs (e.g., competencies, milestones, EPAs), residency programs continue to struggle with the use of milestones and/or EPAs in their curricular goals, objectives, and observational evaluative tools (4) – and little related information has been reported specific to SBP. Hence, we explored how SBP is being integrated into residency education and whether or SBP measures were in alignment with the Next Accreditation System (NAS). We began by reviewing our own internal medicine residency program, and found that our SBP curriculum and its assessments measured associated knowledge and attitudes – but probably not true competency (7). It was obvious, then, that we needed to evaluate in far more greater detail the six components of SBP as they apply to internal medicine residency programs. To move from process measures to hard outcomes like SBP-related developmental milestones or EPAs, we conducted an extensive review of the literature on SBP in internal medicine residency programs. The results of that literature review are presented here, along with recommendations on how to achieve competency and best use NAS evaluative measures in SBP for graduate medical trainees.

## Methods

### Search strategy

**Fig. 1:**
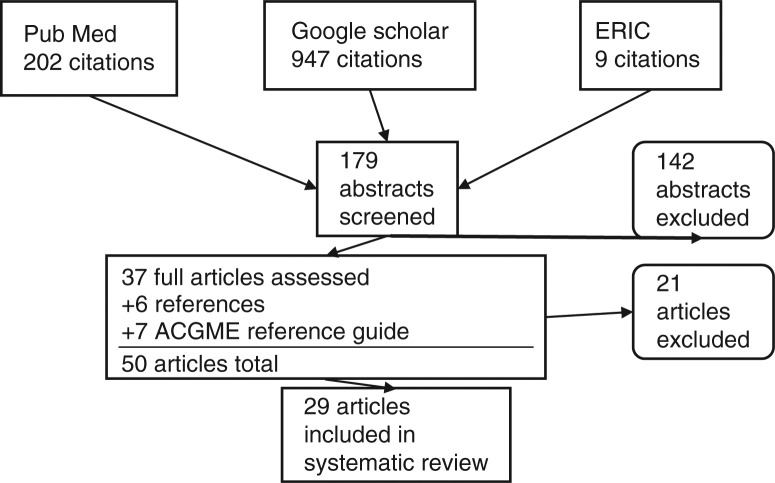
Search strategy for articles on systems-based practice.

We reviewed literature published from 1999 to April, 2012 present using PubMed, Google Scholar, and ERIC (Fig. 1) – using the following key search terms: 1) core competencies; 2) system-based practice; and 3) graduate medical education. For this search 1,158 titles were generated. Of the original 1,158 titles reviewed, 179 titles remained after duplicates, books and titles that named a specialty other than internal medicine were eliminated. The 179 respective abstracts were reviewed and 37 full articles were chosen after applying our inclusion criteria. To these 37 articles, 6 more articles, gleaned from a bibliographic review, were added. We also supplemented our pool with seven articles from the ACGME's Outcomes Project web-based reference guide. After carefully reviewing these 50 articles, we excluded 21 which, despite their titles, did not align with our predetermined inclusion criteria.

### Inclusion and exclusion criteria

All abstracts and articles were included if they contained either a description of the curriculum and/or the associated evaluation methods. The described curriculum had to be focused on the core competency of SBP within US internal medicine residencies. Articles were excluded if they: 1) were in foreign medical programs; 2) were primarily intended for medical students/fellows; 3) focused on more than two core competencies; or 4) involved learners in other (non-internal medicine) specialties. Articles discussing SBP and one other core competency (patient care, medical knowledge, PBLI, professionalism, or interpersonal skills and communication) were included. However, opinion pieces, review articles, and consensus conferences were excluded – as were papers from non-peer-reviewed journals.

### Articles

As stated, the initial search resulted in 1,158 references (Fig. 1). After the above search strategies, removal of duplicates, and a thorough screening using inclusion and exclusion criteria, 29 articles were included in the systematic review (8–36). Given the exploratory nature of this study, we had little basis for organizing these studies (5). A subsequent review of the literature helped define SBP and its principles and implied abilities – from which six categories were developed based on Graham's taxonomy of SBP (1) (Table 1). The primary aim of the reviewers, then, was to categorize each article into one of the six groups; however, several articles contained no single primary theme, but instead meaningfully addressed more than one of the six SBP principles and were subsequently grouped under more than one category. When this occurred, all three authors agreed to do so.

**Table 1:** Categories from SBP definition

ACGME definition	Category/principle	Examples/topics
Work effectively in various health care delivery settings and systems relevant to their clinical specialty	System consultant	Didactics on insurance, managed care, community resources, or experiences in different settings like nursing homes, hospice and/or senior centers
Coordinate care within the healthcare system relevant to their clinical specialty	Care coordinator	Reviewing prior records, scheduling appointments
Incorporate considerations of cost awareness and risk benefit analysis in patient and/or population-based care, as appropriate	Resource manager	Billing, coding, cost containment, care management
Advocate for quality patient care and optimal patient care systems	Patient advocate	Policy, patient education, translators
Work in interprofessional teams to enhance patient safety and improve patient care quality	Team collaborator	Multi-disciplinary rounds- pharmacy, nutrition etc. Enhanced communication skills
Participate in identifying system errors and implementing potential solutions	System evaluator	QI projects, PDSA cycles, sign-outs

A quality index, based on definitions from the ACGME's requirements for achieving competency, was established to ‘grade’ articles. This index ranged from A to D (with A being the highest) and was applied to descriptions of the curriculum and evaluation methods. Because our literature review went back to 1999, the grading system did not include the criteria for milestones or EPAs – which emerged much later (3, 4). The definition of each grade is listed below – and centers primarily on the measured focus of the curriculum and evaluation:

1. Patient outcome(s);
2. Behavioral change by the provider (mostly closely linked to milestones or EPAs);
3. Perceptions, knowledge, or attitudes of providers and/or patients; and
4. Description of content only – no evaluation was completed.

Initial classification of category and quality was done independently by two of the authors (JM and EP). A third reviewer (CH) was used to reconcile disagreements. All reviewers are faculty members involved in residency education. One (JM) is a core faculty member who co-directs two residency educational units, whereas the other (EP) leads the primary care research block. The third reviewer (CH) is the primary care program director.

## Results

After systematically culling the larger sample, the systematic review included 29 articles (Fig. 1). From these, the two reviewers exhibited 66 (19/29) and 90% (26/29) agreement on their assignment of category and grade, respectively.

Only one internal medicine graduate curriculum, represented in 2 of the 29 articles (7%), incorporated all the six principles of SBP into their described curriculum; eight (28%) articles included curricula covering more than one aspect of SBP in a meaningful way. The majority of articles (45%, or 13/29) were classified as ‘system evaluator’ – concentrating on the principle of ‘identifying system errors and implementing potential solutions’, or what is most readily labeled as quality improvement (Table 2). Few articles formally addressed coordination of care or patient advocacy.

**Table 2:** SBP article categorization

Category	Total% (*n*=29)^a^	Article
System consultant	31% (9)	Allen E et al. (2005), David R and Reich L (2005), Eskildsen M (2010), Hingle S et al. (2009), Hingle S et al. (2011), Nagler A et al. (2010), Peters A et al. (2008), Tartaglia K et al. (2010), Turley C et al. (2007)
Care coordinator	7% (2)	Hingle S et al. (2009), Hingle S et al. (2011)
Resource manager	28% (8)	Crites G and Schuster R (2004), Englander R et al. (2010), Hingle S et al. (2009), Hingle S et al. (2011), Korn L et al. (2003), Kravet S et al. (2001), Nagler A et al. (2010), Perez J et al. (2009)
Patient advocate	10% (3)	Hingle S et al. (2009), Hingle S et al. (2011), Tartaglia K et al. (2010)
Team collaborator	31% (9)	Daniel D et al. (2009), Eiser A and Connaughton-Storey J (2008), Hingle S et al. (2009), Hingle S et al. (2011), Kirsh A and Aron D (2006), Nabors C et al. (2011), Sehgal N et al. (2008), Sherman S et al. (2007), Ziegelstein R and Fiebach N (2004)
System evaluator	45% (13)	Allen E et al. (2005), Amin A and Rucker L (2004), Daniel D et al. (2009), Gakhar B and Spencer A (2010), Hingle S et al. (2009), Hingle S et al. (2011), Leenstra J et al. (2007), Oyler J et al. (2008), Peters A et al. (2008), Reznek M et al. (2010), Tomolo A et al. (2005), Wittich C et al. (2010), Zupancic M et al. (2010)

a The % of articles that covered that category.

Only 7% (2/29) were graded A – the highest quality. These articles represented the ACGME's ‘gold standard’ of assessing mastery of a competency via patient outcomes. These two articles reflect examples of what most educators set out to achieve when developing curricula – and go beyond the typical assessment of learners’ knowledge of content. Interestingly, none of the articles, even those published after 2009, stated specifically how milestones and/or EPAs were addressed in their curricular design and/or evaluation process, if they occurred. However, both of the two ‘A’ articles were among those more recently written – suggesting that educators may be refining curricula to mirror more closely the goals of the Outcomes Project and NAS (Table 3).

**Table 3:** Quality index grade of reviewed literature

Grade	Total% (*n*=29)	Article
A^a^	7% (2)	Daniel D et al. (2009), Englander R et al. (2010)
B^b^	24% (7)	Gakhar B and Spencer A (2010), Hingle S et al. (2011), Leenstra J et al. (2007), Nabors C et al. (2011), Oyler J et al. (2008), Sherman S et al. (2007), Zupancic M et al. (2010)
C^c^	55% (16)	Allen E et al. (2005), Crites G and Schuster R (2004), David R and Reich L (2005), Eiser A and Connaughton-Storey J (2008), Kirsh A and Aron D (2006), Kravet S et al. (2001), Korn L et al. (2003), Nagler A et al. (2010), Perez J et al. (2009), Peters A et al. (2008), Reznek M et al. (2010), Sehgal N et al. (2008), Tartaglia K et al. (2010), Tomolo A et al. (2005), Turley C et al. (2007), Ziegelstein R and Fiebach N (2004)
D^d^	14% (4)	Amin A and Rucker L ( 2004), Eskildsen M (2010), Hingle S et al. (2009), Wittich C et al. (2010)

a The described curriculum and its evaluation measure assessed an actual change in patient outcomes.

b The described curriculum and its evaluation measure assessed an actual behavioral change amongst the provide.

c The described curriculum and its evaluation measure assessed a provider and/or patient perception, attitude or knowledge change.

d Description of curriculum alone.

### Study and search limitations

Our search included only published, peer-reviewed articles – each of which contained its own set of limitations and possible biases. Also, the articles did not use uniform definitions or terms, and exclusively focused on internal medicine residency programs. Lastly, because existing rating criteria had not been previously applied to medical education literature, our development and use of the present coding scheme is somewhat novel and, as such, of unknown validity.

## Discussion

This comprehensive literature review suggests that efforts are underway to incorporate SBP into internal medicine residency education – with isolated examples geared toward evaluating competency in this arena. Yet, the data also indicate that residency programs are lagging behind the Outcomes Project's goal of full integration by 2011 – the largest barrier seemingly to be the design of curricula and operational measures that can independently assess each of the six competencies, even with the inclusion of milestones. This review shows that despite much curricular development, there appears to be little agreement and data regarding best practices in assessing learner competence – especially with regards to SBP and its six core principles. Furthermore, areas that require behavior or performance-based assessment (e.g., milestones, EPAs) are, in contrast to knowledge and written assessments (37), still among the least developed areas.

Before moving ahead to Phase IV (2011 and beyond) of the Outcomes Project, educators must verify that the first three phases have been successfully completed. This review of the literature has shown that in an attempt by the ACGME to make definitions widely applicable to all programs and institutions, there exists ‘lack of enough specificity to move our understanding towards what exactly it is residents need to demonstrate in order to indicate proficiency in SBP’ (38). Each article described different methods and approaches, and the data show that most curricula concentrate efforts on only one of the six principle areas of SBP. As a result, there is much progress to make before SBP can be comprehensively taught and assessed as a core competency.

## Lessons learned and guiding principles

Despite the abovementioned challenges, educators should focus on the practical value of proposed educational changes. In the next steps, as an educational community, we must agree upon a basic model of teaching and assessing each competency that also provides room for divergence and innovation. Keeping in mind the ‘gold standard’ of improved patient care, we must move towards hard outcome measures of competency – for example, documenting milestones and completion of EPAs (39, 40).

We offer four guiding principles that might inform educators when creating or restructuring SBP curricula. The principles were applied to our own SBP program efforts (5), but can just as easily be used in any curriculum that will address any of the six core competencies of patient care, medical knowledge, PBLI, SBP, professionalism, and interpersonal skills and communication.

1. The curriculum must include all core principles of the specified competency.
  In a hypothetical example of reformatting a SBP curriculum, the knowledge content should first be reviewed and grouped into its six core, defining principles (i.e., system consultant, care coordinator, resource manager, patient advocate, team collaborator, and system evaluator). If any core principle is not represented, additional material needs to be created to fill the gap.
2. The curricular objectives should be driven by clinical outcomes.
  After categorizing the content, examples of clinical behaviors or patient outcomes can be linked accordingly. The categorization should align with not just the six basic principles of SBP but also the 21 milestones (3) that map to the core competency of SBP (Table 4). For example, being a competent *system consultant* means being able to navigate your patient through a complete Medicaid application or to assist an elderly patient choose a prescription drug plan under Medicare Part D. Assistance with this real clinical experience could map to the milestone of ‘Reflect awareness of common socioeconomic barriers that affect patient care’ or, involving the social worker to complete this task, to the milestone of ‘appreciate role of various health care providers … including social workers’. Under *team collaborator*, a resident may consult with a diabetic nutritionist to create a culturally tailored diet that helps improve a diabetic's glucose control. The examples would then be listed for each category of SBP and its corresponding milestone (Table 4).
3. The teaching modalities should be interactive and clinically relevant.
  The concrete examples outlined above should be incorporated into a curriculum via a teaching strategy that is interactive and clinically based. Some didactic sessions may be warranted, but other pedagogies should be considered. Of course, the degree of innovation will depend on institutional resources, but educational technology such as podcasts (if available) can often overcome scheduling or time barriers. Other examples include case-series, peer-assisted learning, simulations, and direct observations. The teaching and learning modality should also inform the evaluation process.
4. The assessment of competency should utilize milestones and/or EPAs.
  Attention to the course evaluation should be turned from content-oriented to assessment-based, and evaluative tools should focus on patient outcomes or physician behaviors – such as chart reviews, patient satisfaction, or Plan, Do, Study, Act (PDSA) cycles. For example, in addressing the ‘resource manager’ component, one could conduct chart reviews to see how often residents are asking about patients’ abilities to manage the costs of medications and, when a problem is identified, how the resident addressed the issue. For the ‘team evaluator’ aspect, a 360 degree evaluation of a resident involving ancillary staff, social workers, and nurses could be conducted to measure specific physician behaviors toward members of the interprofessional team. Other assessment tools that could be considered are direct observation of SBP skills – such as observing, scoring, and documenting resident–patient encounters around the use of interpreters. There are many other examples; emphasis should remain on curricular objectives driving by clinical outcomes and tied to a milestone (Table 4) or EPA.

**Table 4:** Linking SBP category to milestones

SBP category/principle	Milestone
System consultant	1. Understand unique roles and services provided by local health care delivery systems.
Care coordinator	2. Manage and coordinate care and care transitions across multiple delivery systems, including ambulatory, subacute, acute, rehabilitation, and skilled nursing.
Resource manager	3. Reflect awareness of common socioeconomic barriers that impact patient care.
	4. Understand how cost-benefit analysis is applied to patient care (i.e., via principles of screening tests and the development of clinical guidelines).
	5. Identify the role of various health care stakeholders including providers, suppliers, financiers, purchasers, and consumers and their varied impact on the cost of and access to health care.
	6. Understand coding and reimbursement principles.
	7. Identify costs for common diagnostic or therapeutic tests.
	8. Minimize unnecessary care including tests, procedures, therapies, and ambulatory or hospital encounters.
	9. Demonstrate the incorporation of cost-awareness principles into standard clinical judgments and decision making.
	10. Demonstrate the incorporation of cost-awareness principles into complex clinical scenarios
Patient advocate	11. Negotiate patient-centered care among multiple care providers.
Team collaborator	12. Appreciate roles of a variety of health care providers, including but not limited to consultants, therapists, nurses, home care workers, pharmacists, and social workers.
	13. Work effectively as a member within the interprofessional team to ensure safe patient care.
	14. Consider alternative solutions provided by other teammates.
	15. Demonstrate how to manage the team by using the skills and coordinating the activities of interprofessional team members.
System evaluator	16. Recognize health system forces that increase the risk for error including barriers to optimal patient care.
	17. Identify, reflect on, and learn from critical incidents such as near misses and preventable medical errors.
	18. Dialogue with care team members to identify risk for and prevention of medical error.
	19. Understand mechanisms for analysis and correction of systems errors.
	20. Demonstrate ability to understand and engage in a system-level quality improvement intervention.
	21. Partner with other health care professionals to identify, propose improvement opportunities within the system.

## Conclusion

Many of the lessons learned in reformatting our own SBP curriculum centered on best delivery of teaching methods and assessment tools – both of which should align with current changes in health care and health professions education. Despite not yet being able to share successful experiences from our reformatted curriculum (7), sharing lessons learned may assist other programs in enhancing or creating their own SBP curriculum as mandated by the ACGME. We believe our experience, this literature review, and the guiding principles provided can assist in the development of competency-based curricula.
